# Implementing Multimodal Hardware Security with 2D α‐In_2_Se_3_ Ferroelectric Transistor

**DOI:** 10.1002/advs.202502286

**Published:** 2025-04-15

**Authors:** Xinwei Zhang, Jiachao Zhou, Yishu Zhang, Jian Chai, Yongqing Bai, Hailiang Wang, Qian He, Xi Wang, Lin Wang, Yuda Zhao, Yang Xu, Bin Yu

**Affiliations:** ^1^ College of Integrated Circuits Zhejiang University Hangzhou Zhejiang 311200 China; ^2^ ZJU‐Hangzhou Global Scientific and Technological Innovation Center Hangzhou Zhejiang 311200 China; ^3^ School of Mechanical Engineering Shanghai Jiao Tong University 800 Dongchuan Road Shanghai 200240 China; ^4^ Joint Institute of Zhejiang University and the University of Illinois at Urbana‐Champaign Zhejiang University Haining Zhejiang 314400 China

**Keywords:** ferroelectric α‐In_2_Se_3_, hardware security, in‐memory computing, in‐memory sensing, multimodal, neuromorphic synapses

## Abstract

Security is a critical challenge in the integrated circuit (IC) industry, yet device‐level hardware security remains largely underexplored. Most existing solutions necessitate modifications to current technology nodes and typically address only a single security threat, leaving them vulnerable to diverse attacks while incurring substantial costs in area, energy, and resources. In this study, an out‐of‐the‐box security solution is proposed that integrates an in‐memory sensing and computing (IMSC) architecture based on α‐In_2_Se_3_ transistor, specifically designed for versatile and multimodal secure applications. By leveraging the unique ferroelectric, optoelectronic, and semiconducting properties of α‐In_2_Se_3_, the study demonstrates the secure transistor's electronic and optoelectronic synaptic behaviors, along with its capability for reconfigurable logic operations. Based on these, the secure transistor successfully implements four key security primitives: anticounterfeiting, watermarking, logic locking, and IC camouflaging in a single‐transistor structure, offering robust protection against counterfeit ICs, intellectual property theft, and reverse engineering. The multimodal secure transistor demonstrates the functional flexibility in addressing various security threats.

## Graphical abstract

A multimodal secure transistor‐integrated in‐memory sensing and computing architecture is demonstrated by leveraging its electronic and optoelectronic synaptic behaviors. Key security primitives, such as anticounterfeiting, watermarking, logic locking, and camouflaging, are implemented within a compact single‐transistor structure, providing a scalable and resource‐efficient solution to address hardware security threats in the era of the Internet of Things.

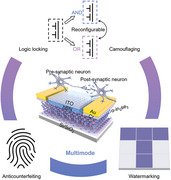

## 1 Introduction

The exponential growth of interconnected devices within the Internet of Things (IoT) and edge computing domains has precipitated critical challenges in hardware security and energy efficiency.^[^ ^1^, ^2^, ^3^ ^]^ In response, the emerging in‐memory sensing and computing (IMSC) architecture represents a transformative approach, integrating sensing, memory, and computational capabilities within a single device.^[^ ^4^, ^5^, ^6^, ^7^ ^]^ By minimizing data movement, IMSC simultaneously reduces latency and power consumption while enabling real‐time processing functions. However, on the other hand, integrated circuits (ICs) are increasingly susceptible to multifaceted threats, including counterfeiting, intellectual property (IP) theft, reverse engineering (RE),^[^ ^8^, ^9^, ^10^ ^]^ which challenge traditional security paradigms. Conventional complementary metal oxide semiconductor (CMOS) technologies encounter significant limitations such as high‐power consumption, area inefficiency, substantial costs, and vulnerability to advanced attacks like machine learning‐driven techniques. Moreover, their reliance on single security mechanism makes it difficult to cope with diverse and evolving attack methods. Therefore, fully integrating hardware security into IMSC designs is indispensable, as security should become a critical consideration alongside performance, reliability, and energy efficiency.

Recent advancements in emerging materials, particularly 2D materials and memristors have introduced unique properties—such as inherent randomness, low power consumption, and dynamic reconfigurability—that could enhance hardware security. These innovations facilitate the development of resilient security primitives, including physically unclonable functions (PUFs),^[^ ^11^, ^12^, ^13^ ^]^ true random number generators (TRNGs),^[^ ^14^, ^15^ ^]^ logic locking (LL),^[^ ^16^, ^17^ ^]^ IC camouflaging,^[^ ^16^, ^18^, ^19^ ^]^ watermarking and anticounterfeiting,^[^ ^19^, ^20^, ^21^ ^]^ addressing the evolving security challenges faced by modern hardware systems. Nonetheless, many existing solutions require complex structures, for example floating gate structure,^[^ ^17^ ^]^ polarity gate design,^[^ ^16^ ^]^ and tend to focus on a single security primitive, rendering them inadequate against emerging threats. Additionally, the implementation of Boolean logic operations through intricate circuit designs,^[^ ^16^, ^17^, ^18^ ^]^ inevitably leads to increased costs, power consumption, and suboptimal area utilization. To address these issues, there is a pressing need for versatile, energy‐efficient devices capable of simultaneously tackling multiple hardware security challenges. Notably, the application of IMSC based on the non‐von Neumann architecture for the realization of various security primitives has not been previously reported, presenting a unique opportunity to consolidate multiple security solutions within a single device framework.

Among the promising candidates for IMSC are ferroelectric semiconductor materials, particularly α‐phase indium selenide (α‐In_2_Se_3_). Its unique combination of ferroelectric, optoelectronic and semiconducting properties enables the realization of IMSC at a simple and compact single‐transistor level without requiring complicated designs such as floating gate or defect engineering. Remarkably, α‐In_2_Se_3_ exhibits both in‐plane (IP) and out‐of‐plane (OOP) ferroelectricity at room temperature with dipole locking effect, even at atomic‐scale thicknesses.^[^ ^22^, ^23^ ^]^ Its moderate bandgap (≈1.39 eV) further enhances its optical performance,^[^ ^24^ ^]^ making it highly suitable for electronic and optoelectronic applications. These exceptional properties position α‐In_2_Se_3_ as an ideal platform for non‐volatile memory, optoelectronic devices and neuromorphic computing.^[^ ^25^, ^26^, ^27^, ^28^ ^]^

In this work, we introduce a novel IMSC architecture using a single‐transistor structure with α‐In_2_Se_3_ as the channel material, presenting a multimodal, low‐power, and area‐efficient hardware security solution. We demonstrate the transistor's excellent gate‐tunable synaptic behavior, characterized by a large memory hysteresis window (≈13.67 V), long‐term retention (>10^4^ s), and remarkable endurance (>10^4^ cycles). By leveraging optoelectronic fusion, we achieve reconfigurable Boolean logic operations—including AND, OR, NAND and NOR—effectively integrating memory, sensing, and computing functionalities within a single transistor. Critically, our multimodal hardware security platform demonstrates four distinct security primitives: anticounterfeiting, watermarking, LL and IC camouflaging. The multimodal hardware security platform we implemented can revolutionize conventional security solutions and promote the advancement of hardware security protection methods in the IoT and edge computing scenarios.

## 2 Results and Discussion

### 2.1 Concept and Characterization of α‐In_2_Se_3_‐Based Secure Transistor

**Figure 1:**
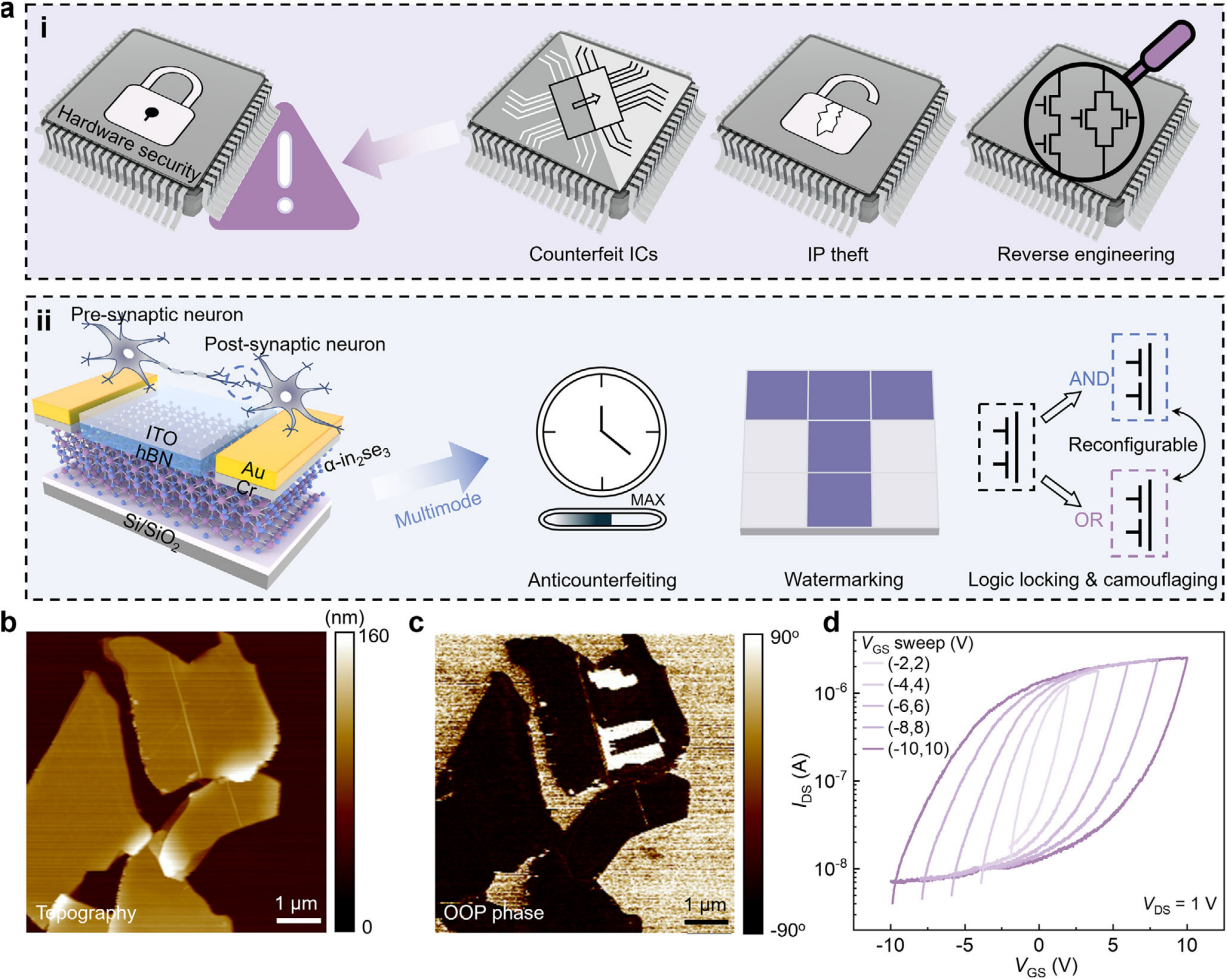
The illustration and characterization of multimodal secure transistor. a(i)) Common threats to hardware security such as counterfeit ICs, IP theft, and RE. a(ii)) Schematic of the IMSC secure transistor, demonstrating four security primitives: anticounterfeiting, watermarking, LL, and IC camouflaging. PFM b) topography, c) OOP phase of α‐In_2_Se_3_. d) Transfer characteristics of a representative device with a clockwise hysteresis loop.

In the current era, the design, manufacturing, and packaging of IoT devices have increasingly become a globally collaborative endeavor. While this trend enhances cost and time efficiency, it has also given rise to significant hardware security threats, such as counterfeit ICs, IP theft, and RE (**Figure** **1**a(i)). Our proposed IMSC secure transistor is illustrated in Figure 1a(ii). This innovative architecture leverages three‐terminal electronic and two‐terminal optoelectronic synaptic properties to implement four distinct security primitives: anticounterfeiting, watermarking, LL and IC camouflaging, thereby effectively enhancing hardware security. The α‐In_2_Se_3_/hexagonal boron nitride (hBN) heterojunction was fabricated by mechanical exfoliation and subsequently transferred onto the Si/SiO_2_ substrate. α‐In_2_Se_3_ was selected as the channel material due to its unique combination of ferroelectric, optoelectronic, and semiconducting properties, while hBN was used as the gate dielectric layer. The source/drain (S/D) electrodes, composed of 10 nm chromium (Cr) and 30 nm gold (Au) were deposited via thermal evaporation, and an indium tin oxide (ITO) gate electrode was deposited using magnetron sputtering. Further details of the fabrication process are provided in the Experimental Section.

Figure S1a (Supporting Information) presents the crystal structure of α‐In_2_Se_3_, which consists of two quintuple layers (QLs) with each quintuple layer following an atomic arrangement of Se–In–Se–In–Se. The Raman spectrum in Figure S1b (Supporting Information) confirms that the α‐In_2_Se_3_ used in this work is the 2H phase (hexagonal structure, *P63/mmc* space group) rather than the 3R phase (rhombohedral structure, *R3* *m* space group), which can be distinguished by the number of stacked QLs. Both 2H and 3R α‐In_2_Se_3_ are non‐centrosymmetric and exhibit OOP and IP ferroelectricity.^[^ ^23^ ^]^ The displacement of the central Se atom in the QL induces simultaneous switching of OOP and IP polarizations, resulting in a dipole locking effect.^[^ ^22^, ^29^, ^30^ ^]^ To characterize the ferroelectric properties of α‐In_2_Se_3_, piezoresponse force microscopy (PFM) was performed on flakes transferred onto a Au substrate. Figure 1b,c shows the topographic and phase images, respectively, revealing clear phase contrasts. Additionally, the hysteresis loops in PFM phase and amplitude as a function of the applied bias voltage exhibit typical behaviors of ferroelectric materials, demonstrating the dynamic process of ferroelectric domain reversal.^[^ ^24^ ^]^ The parallelogram‐shaped piezoelectric response hysteresis and butterfly‐shaped amplitude hysteresis indicate that the ferroelectric polarity is reversed either upward or downward under the write voltage (Figure S2, Supporting Information).

Subsequent electrical measurements were conducted on the device under vacuum conditions. Figure 1d shows the transfer curves obtained from different bidirectional gate voltage sweeps (*V*_GS_), with the S‐D bias (*V*_DS_) fixed at 1 V. A significant clockwise hysteresis is observed, with the hysteresis memory window expanding as the *V*_GS_ sweeping range increases, indicating an accumulation of ferroelectric polarization switching in the channel. This gradual reversal of ferroelectric polarization driven by gate voltage makes it ideally suited for use as an electronic synapse. Figure S3a (Supporting Information) quantifies the memory window of voltage values, revealing a maximum window of ≈13.67 V for a sweeping range of −10 to 10 V. Furthermore, the on/off ratio (*I*_ON_/*I*_OFF_) exceeds 10^2^ under this sweeping range, confirming remarkable electrical performance. Figure S3b (Supporting Information) illustrates the output curves under different *V*_GS_, where hysteresis is evident during the sweep, with the direction indicated by the arrows. To demonstrate that this hysteresis is attributed to the IP polarization of α‐In_2_Se_3_, we performed output characteristic tests at various temperatures and sweeping speeds, as shown in Figure S4 (Supporting Information). It is well‐known that charge trapping process is related to thermal activation, exhibiting strong temperature dependence, and is a much slower process as compared with ferroelectric flipping.^[^ ^31^, ^32^, ^33^ ^]^ The hysteresis windows exhibit slight fluctuations at low temperatures or with fast sweep rates, thus ferroelectric polarization is the dominant factor contributing to the hysteresis. Additionally, we explored the effect of a small *V*_DS_ sweeping range (−1 V, 1 V) on ferroelectric polarization, as presented in Figure S5 (Supporting Information). The dual‐sweep output curve exhibits a negligible hysteresis window. In principle, this behavior is related to the IP ferroelectric coercive voltage, indicating that the IP ferroelectric coercive voltage lies between 1 V and 2.5 V. This also confirms that the observed hysteresis is primarily dominated by IP polarization. Notably, the channel current (*I*_DS_) increases with higher *V*_GS_, suggesting that the IP polarization state is electrically tunable by *V*_GS_.

### 2.2 Three‐Terminal Synaptic Behavior of Gate Electric Stimulation for Anticounterfeiting

**Figure 2:**
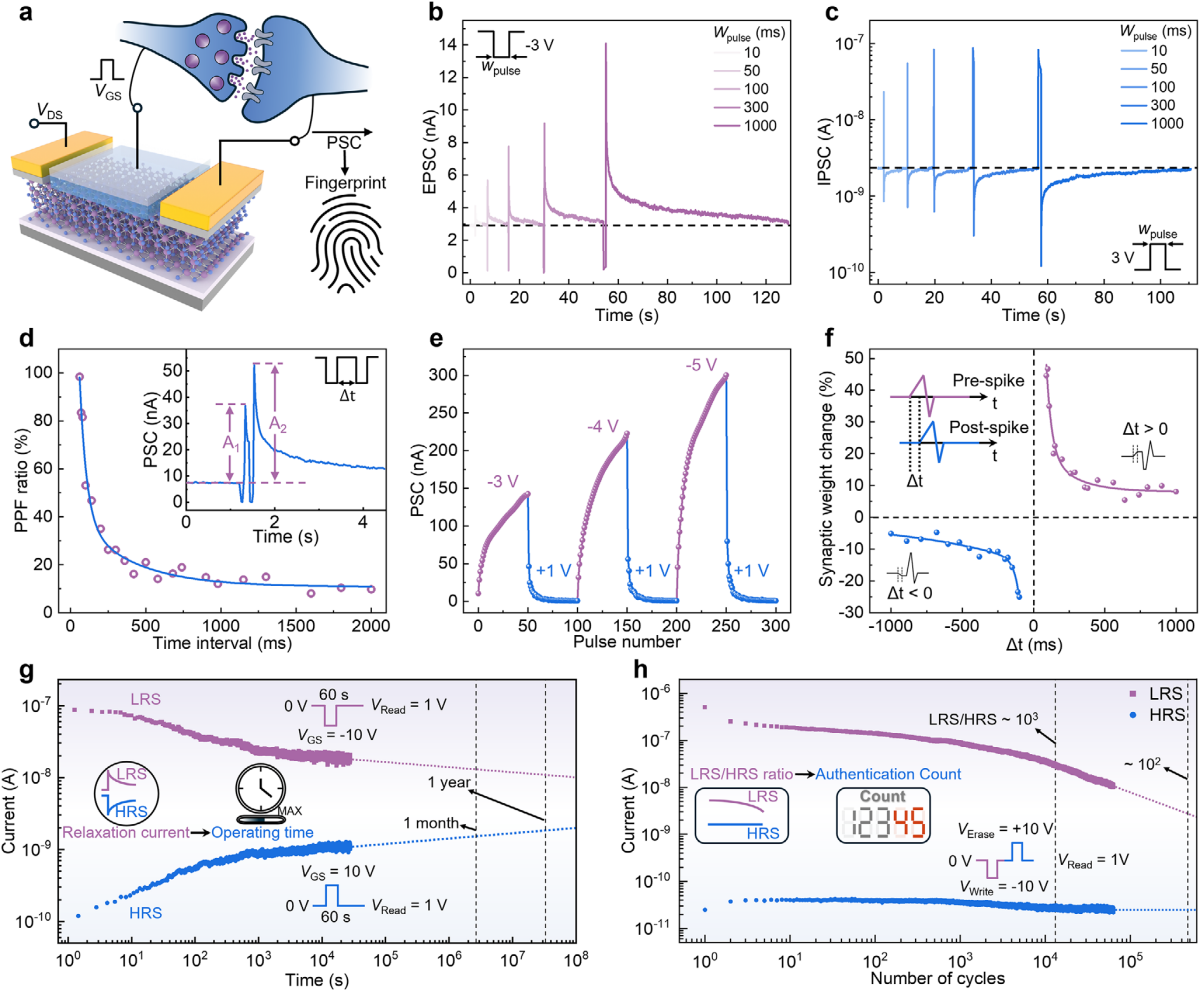
Anticounterfeiting based on gate‐stimulated electronic synaptic behavior of secure transistor. a) A schematic of the electronic synapse, using PSC as an electronic fingerprint to track chip usage. b) EPSC response at different pulse widths under a negative electrical pulse (*V*_GS_ = −3 V), with a read voltage of 1 V. c) IPSC response at different pulse widths under a positive electrical pulse (*V*_GS_ = 3 V), with a read voltage of 1 V. d) PPF ratio as a function of time interval between paired *V*_GS_ pulses (−3 V, 100 ms). The inset shows the typical PSC response following a pair of pulses (−3 V, 100 ms, Δt 100 ms). e) LTP and LTD behaviors of the secure transistor in response to sequential *V*_GS_ pulses with different amplitudes. For LTP, pulse amplitudes of −3, −4, and −5 were applied. For LTD, identical pulses of 1 V amplitude were used. f) Change in synaptic weight as a function Δt between presynaptic and postsynaptic pulses, demonstrating STDP effect. g) Long‐term retention characteristics of the transistor under sufficient write and erase pulses (±10 V, 60 s). By reading the relaxation current, the operating time of the chip can be estimated. h) Endurance characteristics of the device with multiple write and erase cycles (±10 V, 500 ms). By reading LRS/HRS ratio, the authentication count of the chip can be tracked.

The proliferation of counterfeit ICs poses a significant challenge within the semiconductor industry, encompassing issues such as cloning, forgery, recycling, and remarking.^[^ ^8^ ^]^ These counterfeit ICs primarily originate from refurbished electronic waste that re‐enters the market, leading to potentially catastrophic consequences as they typically fail to meet required performance standards. Anticounterfeit measures offer an effective defense against these threats. However, hardware‐based anticounterfeit solutions remain in their infancy and lack robust methods for comprehensive protection. Here, we introduce the concept of an electronic fingerprint, characterized by the postsynaptic current (PSC) of our secure transistor following gate electric stimulation (**Figure** **2**a). Previous studies have shown that the relaxation curve of PSC follows an exponential function,^[^ ^34^ ^]^ which is influenced by both the pulse voltage and pulse width. Due to device‐to‐device (D2D) and cycle‐to‐cycle (C2C) variations, the relaxation curves of PSC are nearly impossible to replicate precisely, even under identical pulse conditions, establishing the PSC as a unique electronic fingerprint for each device.

We first investigated the three‐terminal synaptic behavior under gate electric stimulation, as illustrated in Figure 2a, where the *V*_GS_ serves as the presynaptic input, and the PSC represents the postsynaptic output. Figure 2b shows the current response to increasing pulse widths (*W*_pulse_ = 10, 50, 100, 300, 1000 ms) under a negative electrical pulse (*V*_GS_ = −3 V), with a read voltage of 1 V. Upon application of the negative voltage pulse, the excitatory postsynaptic current (EPSC) initially increases rapidly before gradually relaxing back to baseline, simulating the synaptic short‐term potentiation (STP). Notably, larger pulse widths result in a more pronounced EPSC response. Conversely, Figure 2c presents the current response to increasing pulse widths (*W*_pulse_ = 10, 50, 100, 300, 1000 ms) under a positive electrical pulse (*V*_GS_ = 3 V). Here, the inhibitory postsynaptic current (IPSC) decreases rapidly and then gradually returns back to its initial state, simulating the synaptic short‐term depression (STD). Larger pulse widths similarly result in a more pronounced inhibitory effect. However, if the pulse voltage or width continues to increase, the PSC cannot fully relax to its initial state within a short time. Figure S6 (Supporting Information) extracts the peak current under pulse voltages from ±1 V to ±6 V and pulse widths from 10 to 1000 ms, with corresponding relaxation time extending from several seconds to several minutes, indicating a transition from STP/STD to long‐term potentiation (LTP)/long‐term depression (LTD). The energy consumption (E) per spike can be calculated as *E*  = *V_DS_* × *I* × *t*, where *I* represents peak value in the PSC and *t* is pulse width. When a fixed *V*_DS_ of 1 V is applied, the energy consumption per spike for excitatory (−1 V, 10 ms) and inhibitory (1 V, 10 ms) synapses are 34 and 16 pJ, respectively, which is comparable to biological synapses, demonstrating its potential for low‐power operation. Paired‐pulse facilitation (PPF) as a fundamental characteristic of short‐term plasticity describes the ability of synapses to enhance responses to two consecutive stimuli.^[^ ^35^ ^]^ The PPF ratio is defined as (A_2_‐A_1_)/A_1_ × 100%, where A_1_ and A_2_ are the amplitudes of PSC after the first and second pulses, respectively. PPF is simulated by applying two consecutive pulses (−3 V, 100 ms) with varying time intervals (∆t), as shown in Figure 2d. PPF ratio exhibits biexponential decay as ∆t increases, effectively emulating the biological PPF characteristics, that is $\mathrm{PPF}\;=A_{0}\;+A_{1}^{\prime }*\exp\left(-\Delta t/t_{1}\right)+A_{2}^{\prime }*\exp\left(-\Delta t/t_{2}\right)$, where *A_1_* ′ and *A_2_* ′ are rapid and slow facilitation magnitudes, *t_1_* and *t_2_* are rapid and slow decay time in PPF. The inset shows the typical PSC response following the application of a pair of consecutive presynaptic pulses with ∆t of 100 ms. In biological systems, the transition from short‐term plasticity to long‐term plasticity can also be achieved through repeated stimulation.^[^ ^36^, ^37^ ^]^ As shown in Figure 2e, applying a pulse sequence of alternating negative (−3 V, −4 V, −5 V, 100 ms) and positive pulses (1 V, 100 ms), results in repeatable and stable LTP and LTD responses. Figure 2f demonstrates spike‐timing‐dependent plasticity (STDP), illustrating how synaptic weight changes depend on the timing difference between presynaptic and postsynaptic spikes.

The long‐term retention characteristics of the secure transistor demonstrate stable non‐volatility under sufficient write (−10 V, 60 s) and erase (10 V, 60 s) spikes, with read voltage at *V*_DS_ = 1 V (Figure 2g). This feature enables tracking of the device's operating time. The dependence of the low resistive state (LRS) and high resistive state (HRS) on time can be well‐fitted by a double exponential decay function, represented as follows:

(1) $$\mathrm{PSC}\;\;\left(\mathrm{LRS}\right)=B_{0}+B_{1}*\exp\left(-\frac{t}{t_{1}}\right)+B_{2}*\exp\left(-\frac{t}{t_{2}}\right)$$

(2)

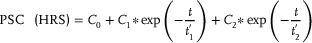

where *B*_1_/*C*_1_ and *B*_2_/*C*_2_ are rapid and slow decay magnitudes, *t*_1_/$t_{1}^{\prime }$ and *t*_2_/$t_{2}^{\prime }$ are rapid and slow decay time, respectively. The values for *t*_1_, *t*_2_, $t_{1}^{\prime }$, and $t_{2}^{\prime }$ are 51, 1428, 179, and 5143 s, respectively, aligning with synaptic timescales. Thus, by simply reading the current value, the age of the chip can be estimated. Owing to the presence of C2C variations, each cycle exhibits distinct peak current values and decay time (Figure S7, Supporting Information). Combined with the lack of information about the pulse voltage and width, it becomes nearly impossible for unauthorized chip recyclers to reset the conductance state to make the chip appear new. In addition to tracking the operating time, the number of uses or successful authentications of the chip is also crucial data for anticounterfeiting. Figure 2h illustrates the device's endurance over multiple write (−10 V, 500 ms) and erase cycles (10 V, 500 ms), revealing a noticeable decay in the LRS after a certain number of cycles, which can be fitted by a double exponential decay function:

(3) $$I\;\;\left(\mathrm{LRS}\right)=D_{0}+D_{1}*\exp\left(-\frac{n}{n_{1}}\right)+D_{2}*\exp\left(-\frac{n}{n_{2}}\right)$$

where *D*_1_, *D*_2_, *n*_1_ and *n*_2_ are constants, with *n*_1_ and *n*_2_ being 688 and 11494, respectively. The fitted curves are shown in Figure S8 (Supporting Information). By associating each successful authentication with one cycle, the LRS/HRS ratio can be used to estimate the number of times the chip has been used. As the chip continues to be used, the secure transistor continuously tracks the total number of usage, as indicated by the LRS/HRS ratio. These electronic fingerprint data—operating time and authentication count—will be stored in a secure database accessible to both developers and users, effectively deterring the recycling and remarking of used ICs. Each hardware module or circuit within the chip can be associated with multiple secure transistors to individually track its specific age and authentication count (D2D variations is shown in Figure S9, Supporting Information). Moreover, multiple secure transistors can monitor usage separately under varied pulse voltages or widths (Figure S10, Supporting Information). This device‐level anticounterfeit scheme does not significantly increase area overhead. We conducted temperature‐dependent retention and endurance tests (Figure S11, Supporting Information), confirming that the performances do not degrade with temperature variations. Additionally, chip manufacturers can embed a maximum permissible operating time and authentication count by predefining threshold currents and LRS/HRS ratios in the electronic fingerprint. Consequently, the chip will cease operation once its age or authentication count reaches the manufacturer's preset limit. This approach not only detects counterfeit ICs but also prevents their operation if they exceed the maximum allowable usage count.

### 2.3 Optical Sensing for Concealable Watermarking

**Figure 3:**
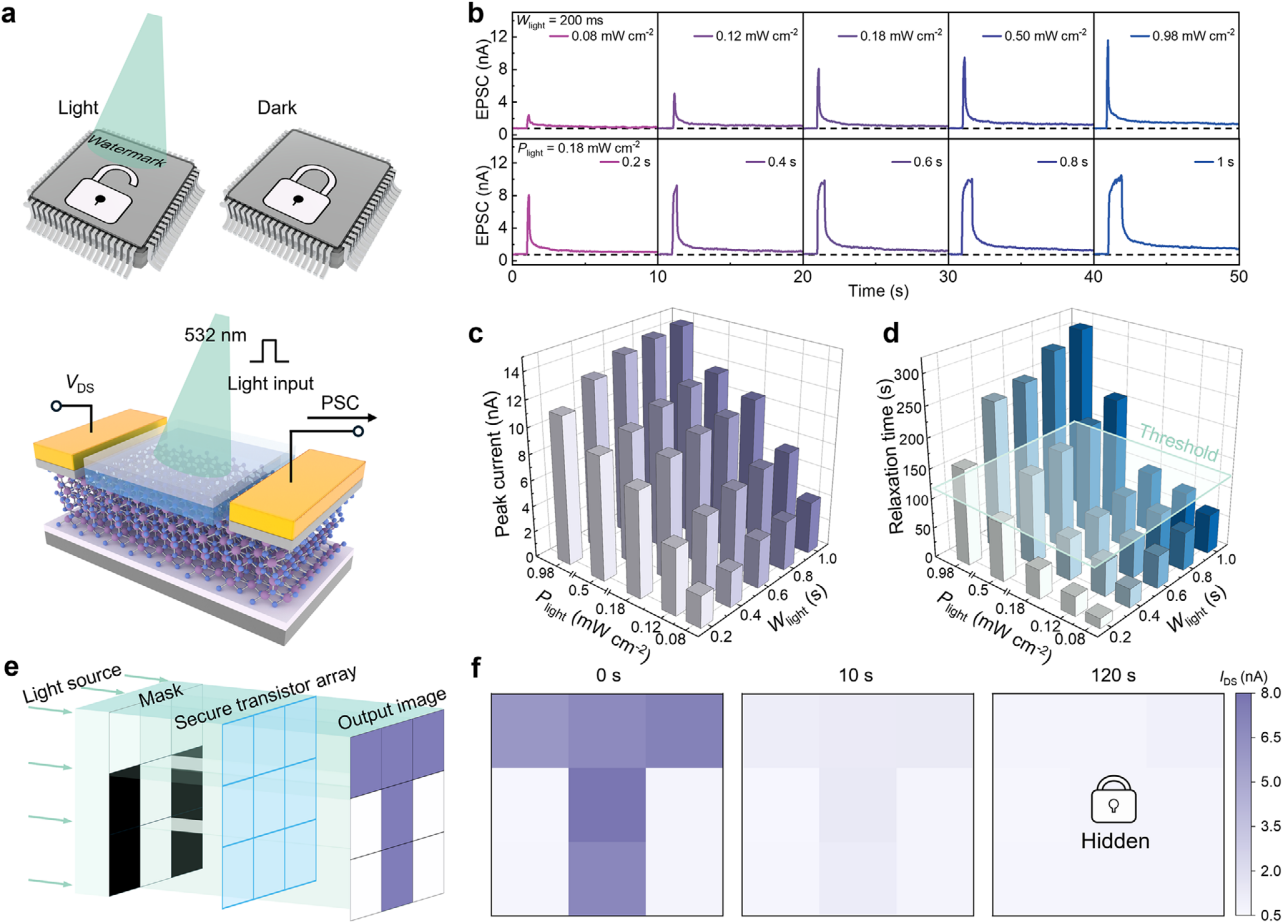
Watermarking based on optoelectronic synaptic behavior of secure transistor. a) Schematic of the watermarking and the optoelectronic synapse. The watermark becomes visible under illumination and fades in darkness. b) Upper part: PSC response under varying optical pulse intensities with a fixed illumination time (200 ms). Lower part: PSC response under varying illumination time with a fixed light intensity (0.18 mW cm^−2^). Extracted c) peak current and d) relaxation time of PSC under different light intensities and pulse widths. e) Schematic diagram illustrating the arrangement of secure transistor array for watermarking. f) Current map of the “T”‐shaped watermark in a 3 × 3 array. Images were captured at 0, 10, and 120 s after the removal of the light stimulus, and the watermark is completely hidden after 120 s. The light intensity in the letter region was set to 0.12 mW cm^−2^, with a pulse width of 1 s.

In addition to counterfeit ICs, IP theft represents a growing threat to the semiconductor industry. Watermarking, which involves embedding a unique, hidden signature within the IP by the designer to assert ownership, is typically absent in pirated copies. While many watermarking methods have been developed at the algorithmic and circuit levels,^[^ ^38^, ^39^, ^40^ ^]^ approaches at the material and device levels remain largely underexplored. Here, as illustrated in **Figure** **3**a, we propose an innovative concealable watermarking technique utilizing the optoelectronic synaptic behavior of the secure transistors, where the light serves as the presynaptic input while PSC acts as the postsynaptic output. The watermark becomes visible under illumination and fades in darkness, effectively preventing IP theft and complicating replicate efforts. To demonstrate this concept, the device was illuminated with a 532 nm laser while a small bias (*V*_DS_ = 1 V) was applied to read the channel current. As light intensity increases (0.08, 0.12, 0.18, 0.50, and 0.98 mW cm^−2^) with a fixed pulse width of 200 ms, the device exhibits an enhanced EPSC (upper part of Figure 3b). This is attributed to the photo‐generated photocurrent, which raises the PSC. In α‐In_2_Se_3_, the photocurrent can be divided into a fast response due to intrinsic photoconductive mechanisms and a slow response resulting from trap‐induced effects.^[^ ^41^ ^]^ Higher light intensities generate more photogenerated carriers, leading to a stronger current response. The lower part of Figure 3b illustrates the current response under varying pulse widths (0.2, 0.4, 0.6, 0.8, and 1 s) with a fixed light intensity of 0.18 mW cm^−2^. Only slight changes in current response are observed, as the consistent light intensity produces a similar level of intrinsic fast response. However, the increased pulse width fills more traps, leading to a marginal increase in current. Figure 3c summarizes the peak current of PSC under different light intensities and pulse widths, with corresponding relaxation time statistics shown in Figure 3d and Figure S12 (Supporting Information). As both light intensity and pulse width increase, the current response transitions from STP to LTP. However, even at a light intensity of 0.98 mW cm^−2^ and a pulse width of 1 s, the PSC recovers to its initial state within approximately 300 s, demonstrating strong potential for creating concealable watermark. We also conducted temperature‐dependent photoresponse tests, as depicted in Figure S13 (Supporting Information). The results indicate that the device exhibits similar photoresponse characteristics across different temperatures, suggesting that the optical properties are intrinsic to α‐In₂Se₃ and do not undergo significant changes with temperature variations.

To provide definitive proof of concept, we constructed a 3 × 3 secure transistor array to illustrate its application in a concealable watermark. This array was exposed using an optical mask shaped like the letter “T”, as shown in Figure 3e. We set a threshold of 120 s (Figure 3d), assuming that an exposed watermark exceeding this threshold may be at risk of theft. In this demonstration, the light intensity in the letter region was set to 0.12 mW cm^−2^, with a pulse width of 1 s. Images were captured immediately after removing the light stimulus, as well as 10 and 120 s later (Figure 3f). After light exposure, the device current increases, making the watermark clearly visible. Due to the STP behavior of the device, the watermark remains observable for a period after the light pulse is removed. Once the PSC returns to its initial state, the watermark becomes completely hidden. We can adjust the relaxation time based on specific application requirements. As presented in Figure S14 (Supporting Information), we set the light intensity in the letter region to 1.02 mW cm^−2^ with a pulse width of 1 s. The watermark remains clearly visible even after 1 min. Our secure transistor provides dual‐layer protection for IP: first, each chip can incorporate an N × N watermark pattern designed by the developer or designer; second, the watermark is only visible under illumination and fades quickly once the exposure is removed, making it unlikely for a reverse engineer to realize that illumination is required to access the watermark.

### 2.4 Reconfigurable Logic Operations for Logic Locking and Camouflaging

RE is another common security threat in the IC industry. Adversaries can readily obtain the layout of an IC chip through various RE techniques, such as transmission electron microscopy (TEM)^[^ ^42^ ^]^ and X‐ray imaging,^[^ ^43^ ^]^ allowing them to decipher the chip's functionality. To counter this threat, hardware security techniques such as LL and IC camouflaging have been developed. LL modifies the IC's behavior so that it does not produce the expected output unless activated with a specific key.^[^ ^44^ ^]^ IC camouflaging, on the other hand, disguises the actual function of a circuit by using standard cells that appear identical but perform different functionalities, effectively concealing the circuit's true operation.^[^ ^45^ ^]^ Even if a reverse engineer manages to map out the entire chip layout, the chip's functionality remains concealed without the correct key, as the individual functions of circuit cell remain unknown. The chip operates correctly only when all the necessary keys are provided during runtime to configure the logic gates to their intended functions.

**Figure 4:**
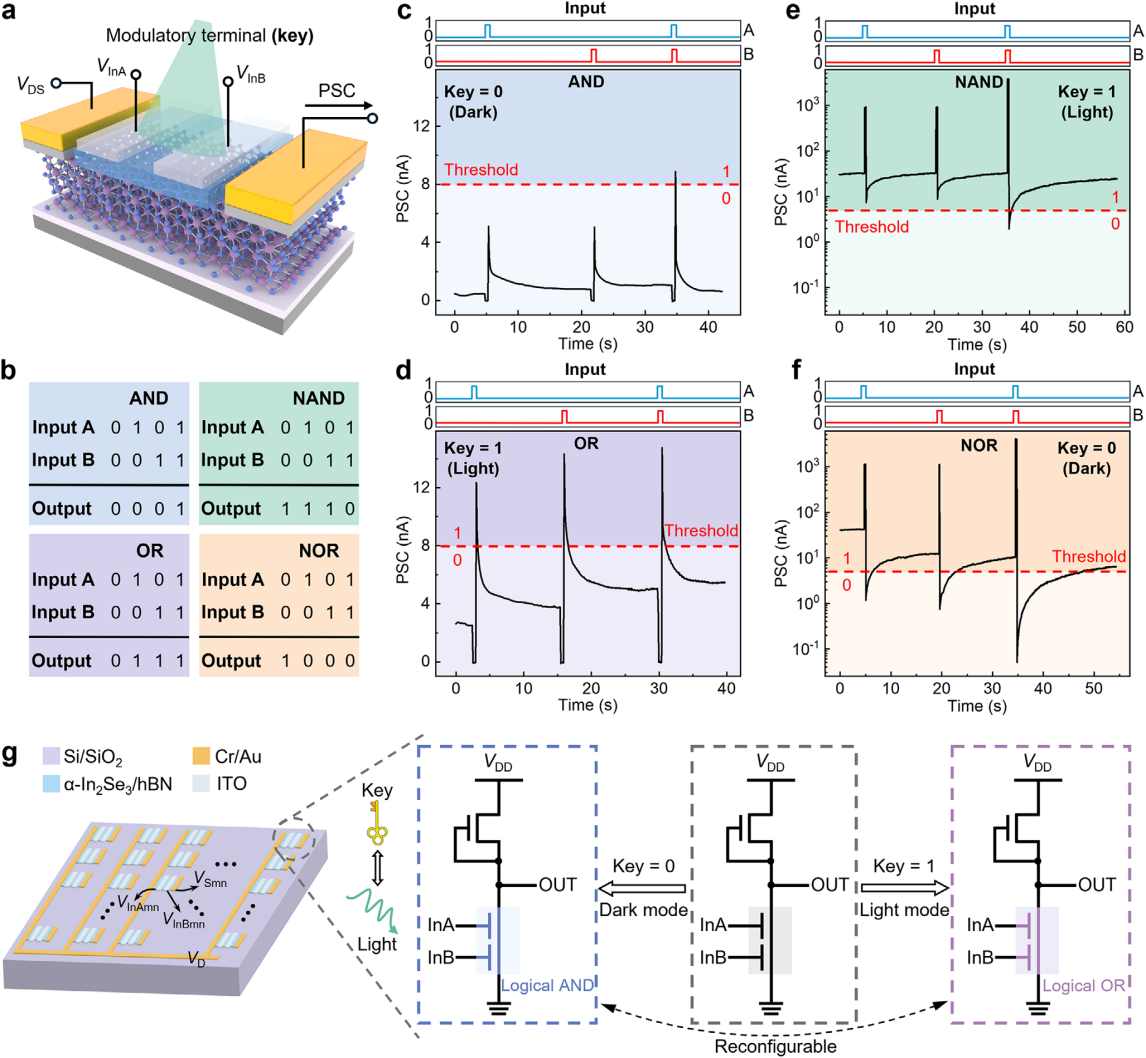
LL and IC camouflaging based on reconfigurable logic operations. a) Schematic of the split‐gate synaptic device. The optical signals are considered as an additional modulatory terminal (the specific key). b) Truth tables of four basic logic gates. c) “AND” function realized by using negative pulses as inputs A and B, with encoding the key as 0. d) “OR” function realized by using negative pulses as inputs A and B and encoding the key as 1. e) “NAND” function realized by using positive pulses as inputs A and B and encoding the key as 1. f) “NOR” function realized by using positive pulses as inputs A and B, with encoding the key as 0. g) A schematic illustrating the implementation of LL and IC camouflaging (using AND/OR as example), where the key is light. When key = 0 (in dark mode), the device operates as AND, while key = 1 (in light mode), it functions as OR. This configuration is reconfigurable.

The results presented in Figures 2 and 3 demonstrate dynamic responses to both electrical and optical stimuli within the same synapse, attributed to the ferroelectric and optoelectronic properties of α‐In_2_Se_3_. As shown in **Figure** **4**a, using a split‐gate synaptic device as the basic building block instead of a traditional transistor, enables the programming of the channel current, providing an additional degree of freedom for applications in both digital and analog circuits (the optical image of a split‐gate device is shown in Figure S15, Supporting Information). The split‐gate electrodes can be used to apply the input voltage (*V*_InA_ and *V*_InB_) during logic operations. At the same time, optical signals (key) serve as an additional modulatory terminal to program the channel current (Output). The truth tables of the four basic Boolean logic functions are shown in Figure 4b. These four logic functions can be used to construct a complete logic system. For logical “AND” and “OR”, the current threshold is set to 8 nA, with the input logic signal 0 or 1 corresponding to no pulse or a negative electrical pulse (−3 V, 500 ms), respectively. Encoding the key as 0, that is, under dark condition (Figure 4c), where no photogenerated carriers are present, the channel current reaches the threshold only if both split‐gates transmit input signal 1, representing the logical “AND”. This is because the ferroelectric polarization switching induced by applying electrical pulses to both gates is stronger than that induced by a single gate, leading to more pronounced excitatory effects. Encoding the key as 1, that is, under light condition (Figure 4d), due to the help of photogenerated carriers, either *V*_InA_ or *V*_InB_ can trigger the output current to exceed the threshold value, demonstrating an “OR” function. Similarly, logic functions of “NAND” and “NOR” are demonstrated by using none or a positive electrical pulse (3 V, 500 ms) as logic signal 0 or 1, with the current threshold set to 5 nA. When the key is encoded as 1 (light condition), the output current falls below the threshold value only when inputs A and B are simultaneously applied (the ferroelectric polarization switching induced by electrical pulses applied to both gates results in a more pronounced inhibitory effect), demonstrating a “NAND” function (Figure 4e). The “NOR” function is illustrated by modulating either *V*_InA_ or *V*_InB_ to trigger the current below the threshold when the key is encoded as 0 (dark condition) (Figure 4f). These reconfigurable logic states open a way to configure secure transistors into a large set of distinct logic circuits. When multiple secure transistors are assembled into a logic gate, the number of possible functions grows exponentially with the number of devices.

Based on this principle, if we consider the logic‐operating devices in a secure IC as our split‐gate synaptic devices, light modulation serves as the key. Here, we use logical AND/OR as an example, as demonstrated in Figure 4g. When the key is 0 (in dark mode), the secure transistor operates in logical AND. When the key is 1 (in light mode), it functions as logical OR. Compared to conventional silicon transistors, our novel logic devices significantly reduce circuit area per functionality while effectively achieving LL and IC camouflaging. Utilizing light as the specific key instead of electricity presents a novel and unexpected approach to enhancing security.

## 3 Conclusion

In conclusion, we present a secure transistor‐integrated IMSC architecture based on α‐In_2_Se_3_, showcasing its potential to enhance hardware security through a single‐transistor design that implements four security primitives. By exploiting the unique ferroelectric, optoelectronic, and semiconducting properties of α‐In_2_Se_3_, we combine the gate‐tunable synaptic behavior of the secure transistor with an electronic fingerprint, utilizing its long‐term retention and endurance characteristics to effectively track the usage history of chips and demonstrate anticounterfeiting capabilities. The optoelectronic synaptic behavior is employed for watermarking, thereby safeguarding IP from theft. Additionally, we exploit the reconfigurable logic operations enabled by the split‐gate structure to implement LL and IC camouflaging, providing robust defenses against RE. Compared to traditional CMOS‐based solutions, our single‐transistor implementation of multiple security primitives offers improved efficiency in terms of power consumption and area utilization. This work not only addresses the growing security demands in IoT and edge computing scenarios but also represents a foundational step toward secure, multimodal, and adaptive electronics, laying the groundwork for future research into integrated and energy‐efficient hardware security systems.

## 4 Experimental Section

### Device Fabrication

The α‐In_2_Se_3_ and hBN bulk crystals were purchased from HQ Graphene Company. 2D nanoflakes were mechanically exfoliated from bulk crystals and dry transferred to fabricate the α‐In_2_Se_3_/hBN heterostructures. Using polydimethylsiloxane (PDMS), the flakes were transferred onto a highly p‐doped Si substrate with thermally grown 285 nm thick SiO_2_ layer, aligned with a homemade micromanipulator. Next, photoresist 5350 was spin‐coated at 4000 rpm and baked on a hot plate at 105 °C for 5 min. The source and drain electrodes were patterned through standard direct writing laser lithography (DWL), followed by thermal evaporation (10 nm Cr/30 nm Au) and a lift‐off process. A second DWL process was used to pattern the gate electrode, followed by magnetron sputtering (30 nm ITO) and a final lift‐off process.

### Materials Characterization

The Raman spectrum of mechanically exfoliated α‐In_2_Se_3_ flakes on the SiO_2_ substrate were obtained using a WITec Alpha 300R Raman Spectrometer with a 532 nm excitation laser. PFM measurements were conducted on flakes exfoliated onto an Au film using a multimode AFM system (Bruker Dimension Icon). The OOP PFM signal was recorded at a drive frequency of 280 kHz and a drive amplitude of 1500 mV.

### Electrical Measurements

Electrical characterization was performed by a Keithley 4200‐SCS semiconductor parameter analyzer with a probe station in vacuum environment at room temperature. The electric and light pulses were generated by a function signal generator (RIGOL DG5101) and a 532 nm laser source.

## Conflict of Interest

The authors declare no conflict of interest.

## Author Contributions

X.Z. and J.Z. contributed equally to this work. B.Y., Y.X., and Y.Z. guided the research. X.Z and J.Z. designed the experiments and fabricated and tested the devices. J.C., Y.B., and X.W. helped with the electrical measurements. L.W. helped with PFM measurements. H.W., Q.H., and Y.Z. helped with data analysis. All authors contributed to interpreting the data and writing the manuscript.

## Supporting information



Supporting Information

## Data Availability

The data that support the findings of this study are available from the corresponding author upon reasonable request.
