# Supplementary material for: Implementing Multimodal Hardware Security with 2D α‐In2Se3 Ferroelectric Transistor
Source: Adv Sci (Weinh). 2025 Apr 15;12(26):2502286. doi: 10.1002/advs.202502286 (PMC12244506; doi:10.1002/advs.202502286)
Supplement: Supplementary file 1 — Supporting Information [file ADVS-12-2502286-s001.docx]

Supporting Information

Implementing Multimodal Hardware Security with Two-Dimensional α-In_2_Se_3_ Ferroelectric Transistor

Xinwei Zhang,^#^ Jiachao Zhou,^#^ Yishu Zhang,* Jian Chai, Yongqing Bai, Hailiang Wang, Qian He, Xi Wang, Lin Wang, Yuda Zhao, Yang Xu,* Bin Yu*

#These authors contributed equally to this work

X. Zhang, J. Zhou, Y. Zhang, J. Chai, Y. Bai, H. Wang, Q. He, X. Wang, Y. Zhao, Y. Xu, B. Yu

College of Integrated Circuits, Zhejiang University, Hangzhou, Zhejiang 311200, China

X. Zhang, J. Zhou, Y. Zhang, J. Chai, Y. Bai, H. Wang, Q. He, X. Wang, Y. Zhao, Y. Xu, B. Yu

ZJU-Hangzhou Global Scientific and Technological Innovation Center, Hangzhou, Zhejiang 311200, China

L. Wang

School of Mechanical Engineering, Shanghai Jiao Tong University, 800 Dongchuan Road, Shanghai 200240, China

Y. Xu

Joint Institute of Zhejiang University and the University of Illinois at Urbana-Champaign, Zhejiang University, Haining, Zhejiang 314400, China

E-mail: zhangyishu@zju.edu.cn; yangxu-isee@zju.edu.cn; yu-bin@zju.edu.cn


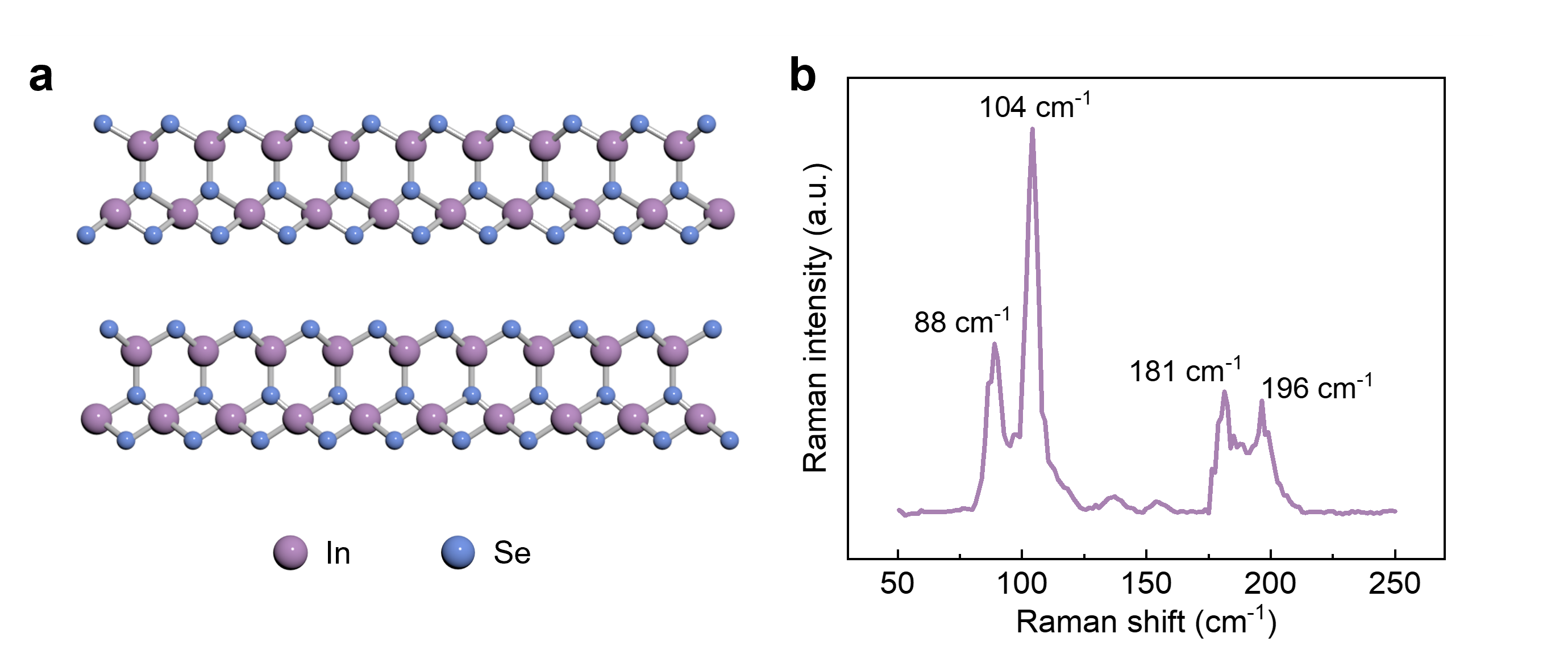


**Figure S1.** a) The crystal structure of α-In_2_Se_3_, where the purple and blue balls represent In and Se atoms, respectively. b) Raman spectrum of α-In_2_Se_3_ flakes, with peaks presented at 88, 104, 181 and 196 cm^−1^.


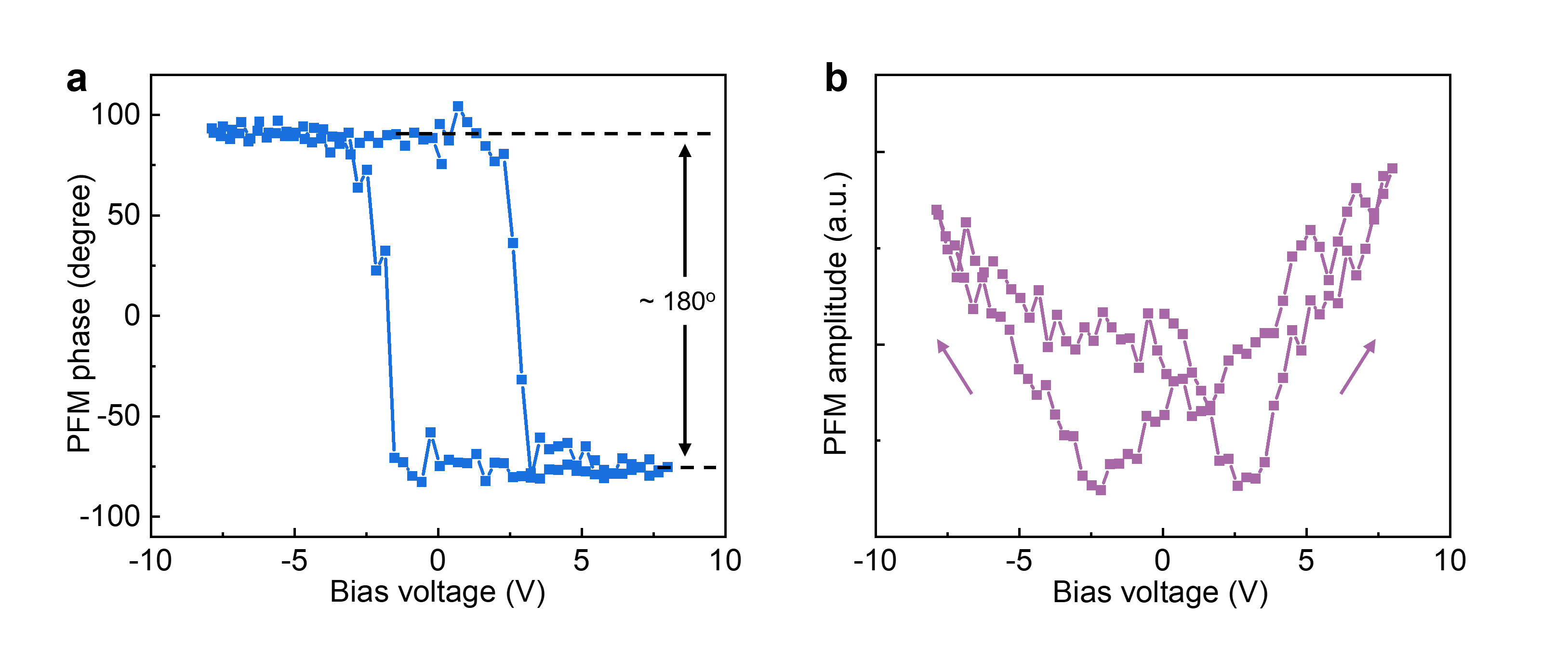


**Figure S2.** Hysteretic loop of OOP PFM a) phase and b) amplitude versus bias voltage on the tip, revealing ferroelectric polarization switching process.


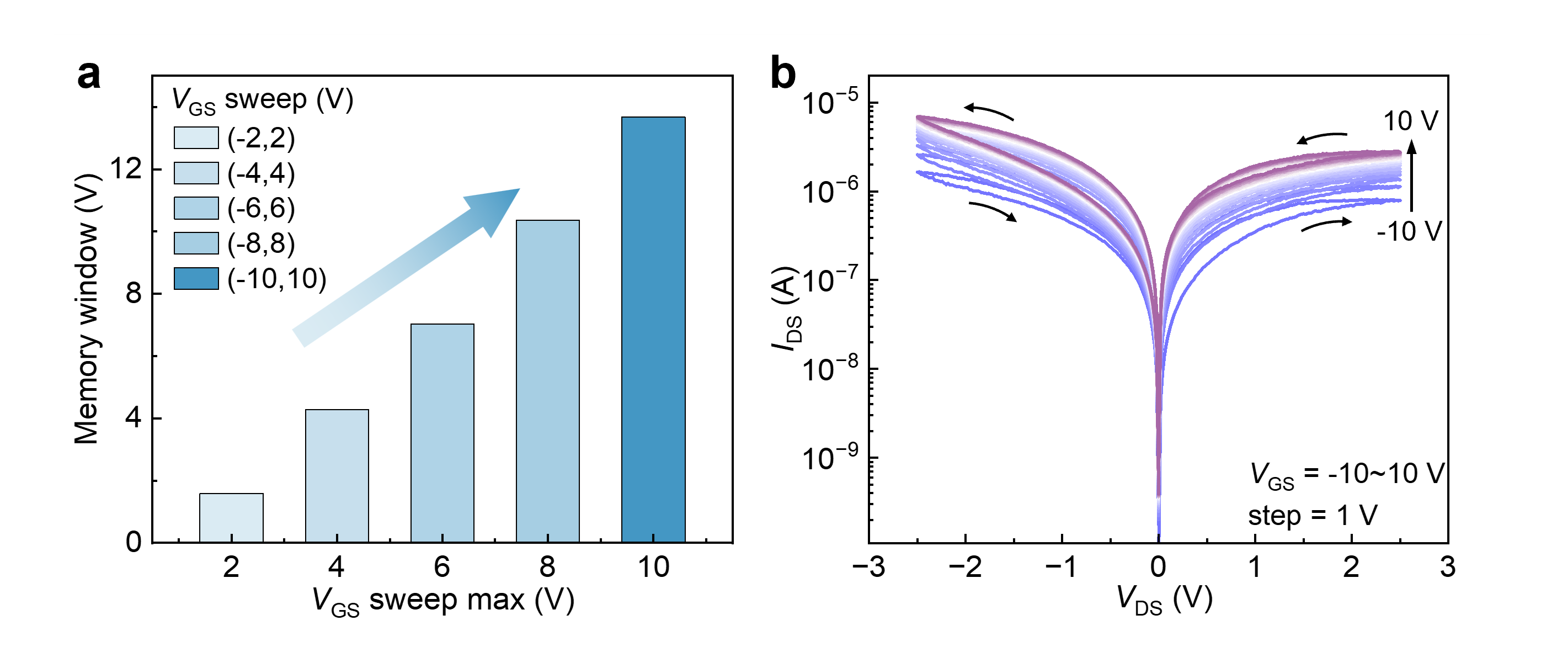


**Figure S3.** a) The memory window extracted in transfer characteristics curves. b) Output characteristics of a representative device with consecutive measurements under different *V*_GS_ bias.


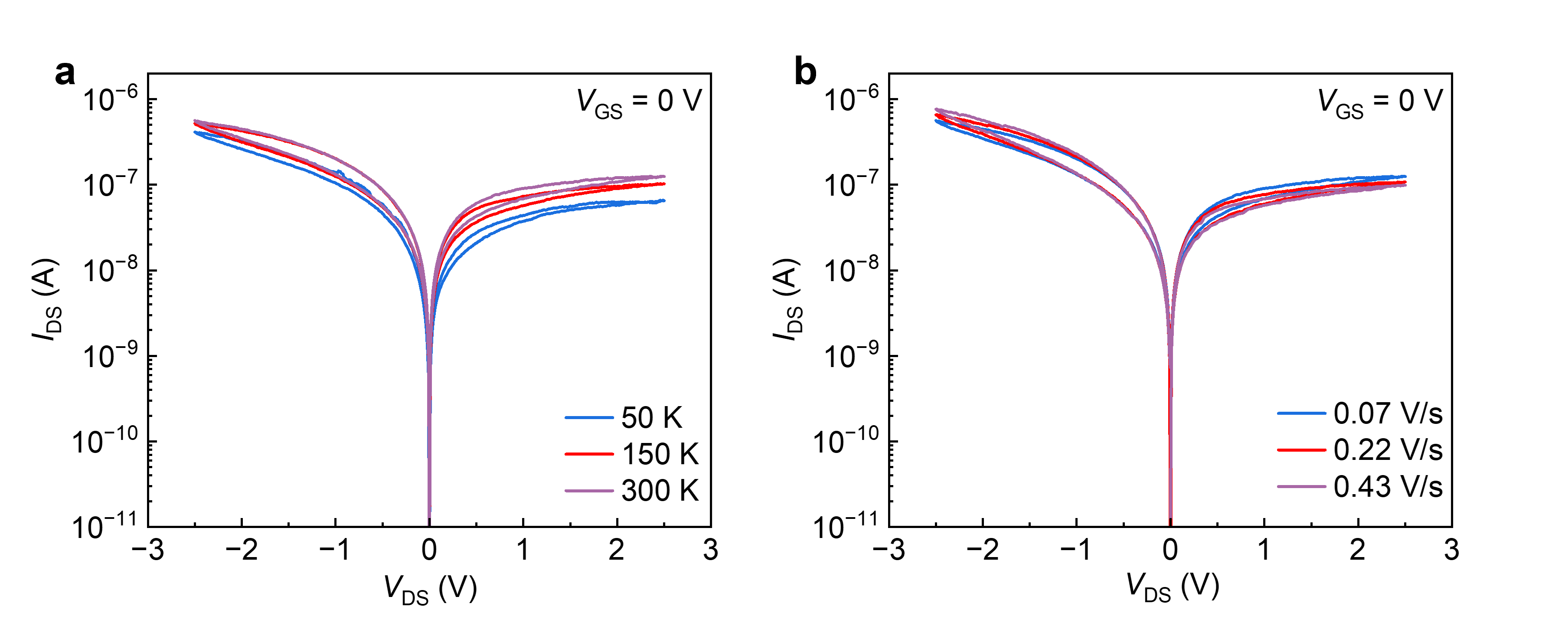


**Figure S4.** The output characteristics of secure transistor at various a) temperatures and b) sweep rates.


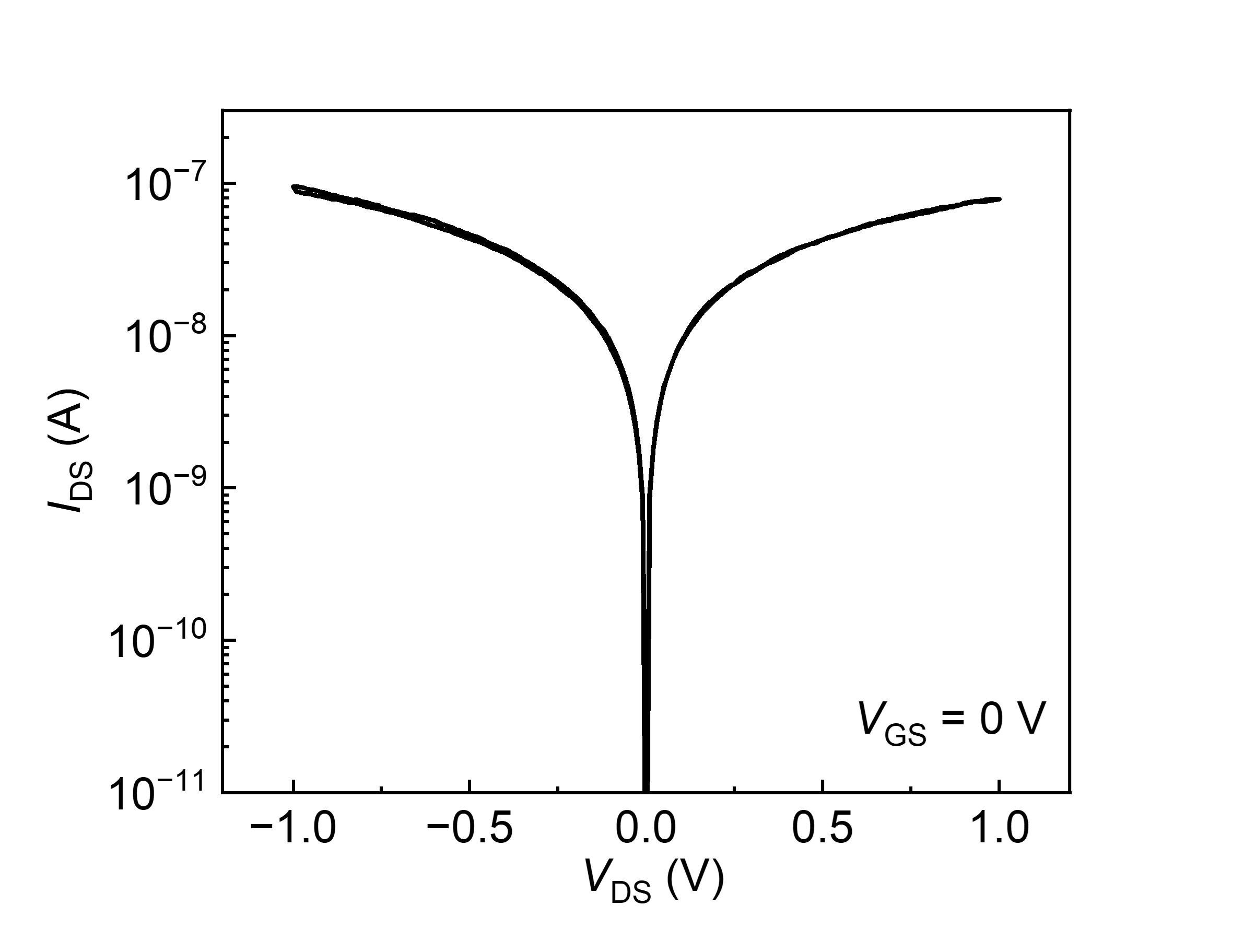


**Figure S5.** The effect of small *V*_DS_ on ferroelectric polarization. The dual-sweep output curve exhibits a negligible hysteresis window, indicating that 1 V is insufficient to reach the IP ferroelectric coercive voltage.


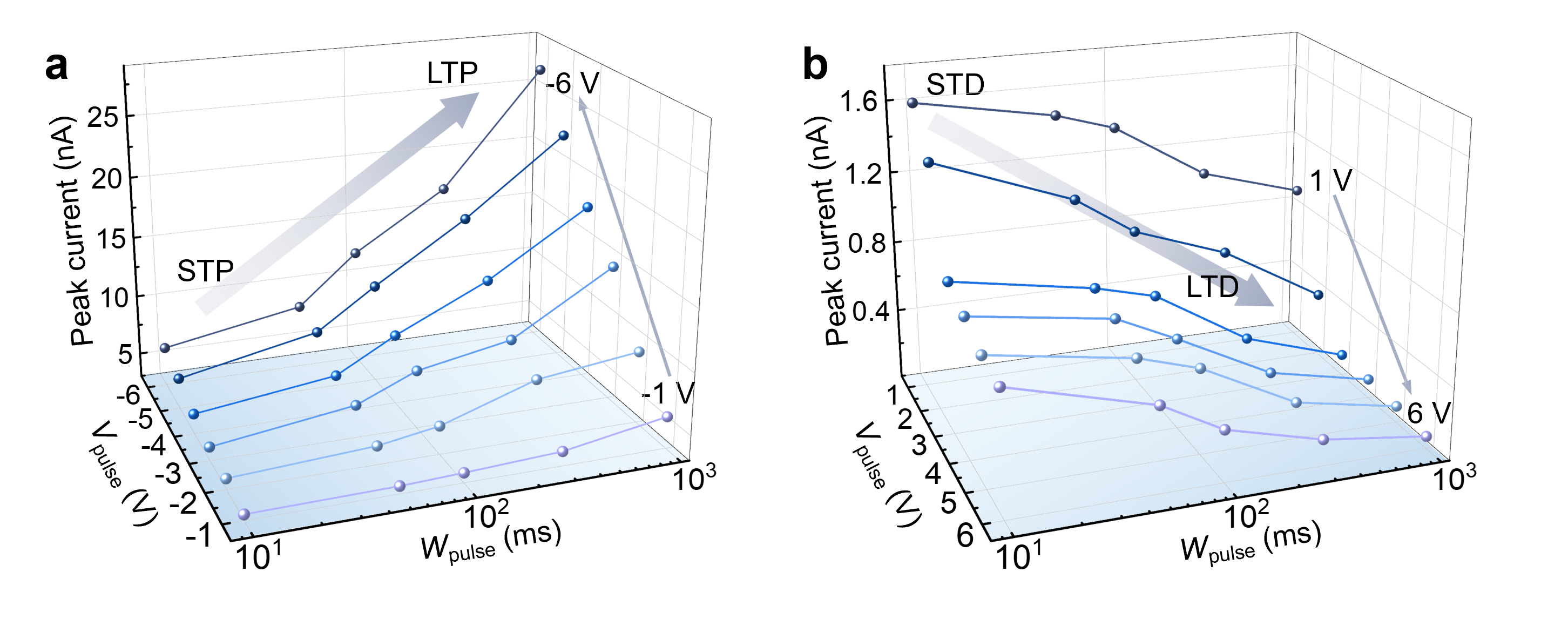


**Figure S6.** a) Extracted peak current under pulse voltages from -1 V to -6 V and pulse widths from 10 ms to 1000 ms, showing the transition from STP to LTP. b) Extracted peak current under pulse voltages from 1 V to 6 V and pulse widths from 10 ms to 1000 ms, illustrating the transition from STD to LTD.


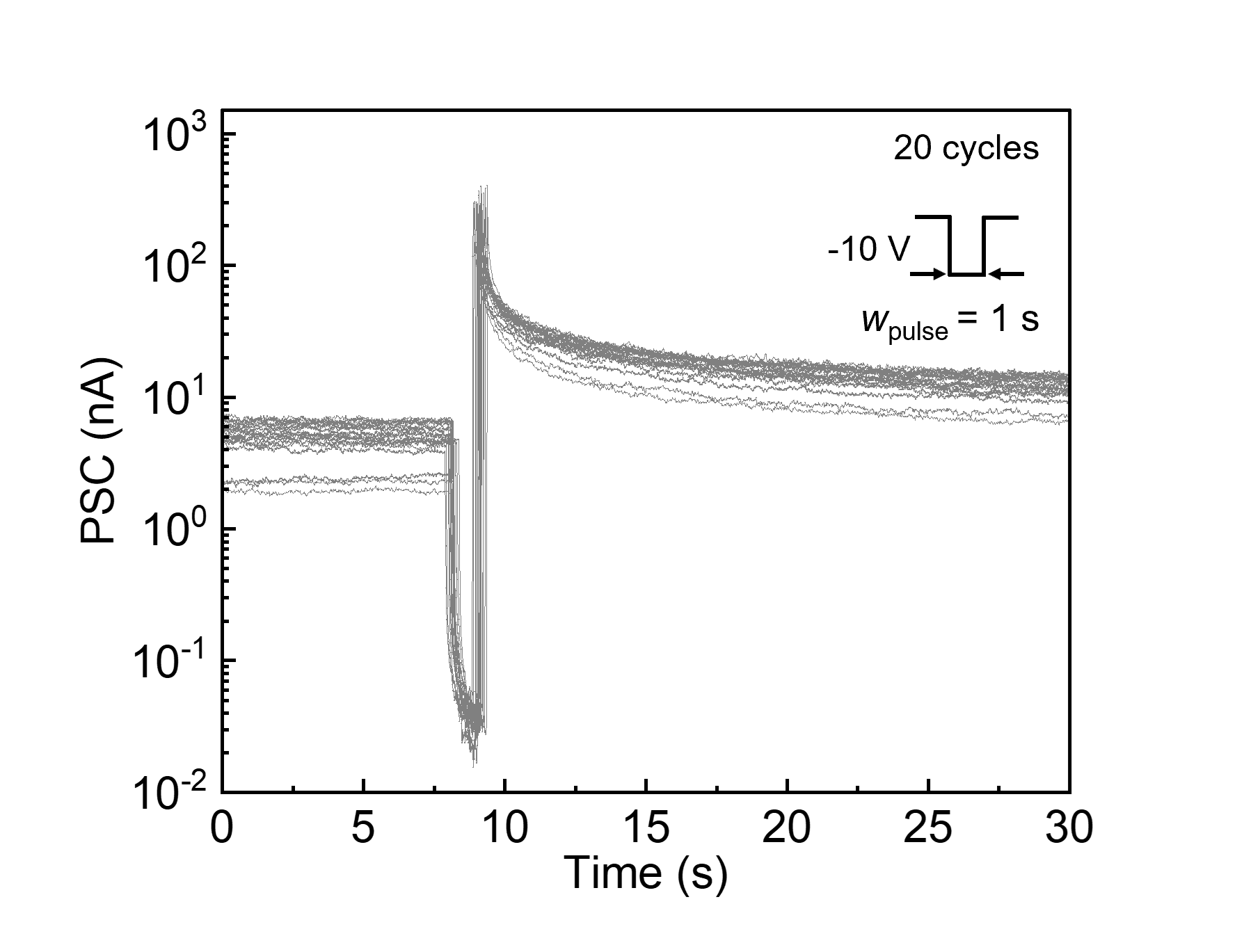


**Figure S7.** Cycle-to-cycle variations of PSC under 20 cycles of identical pulse conditions (-10 V, 1 s), where each cycle exhibits distinct peak current values and decay time.


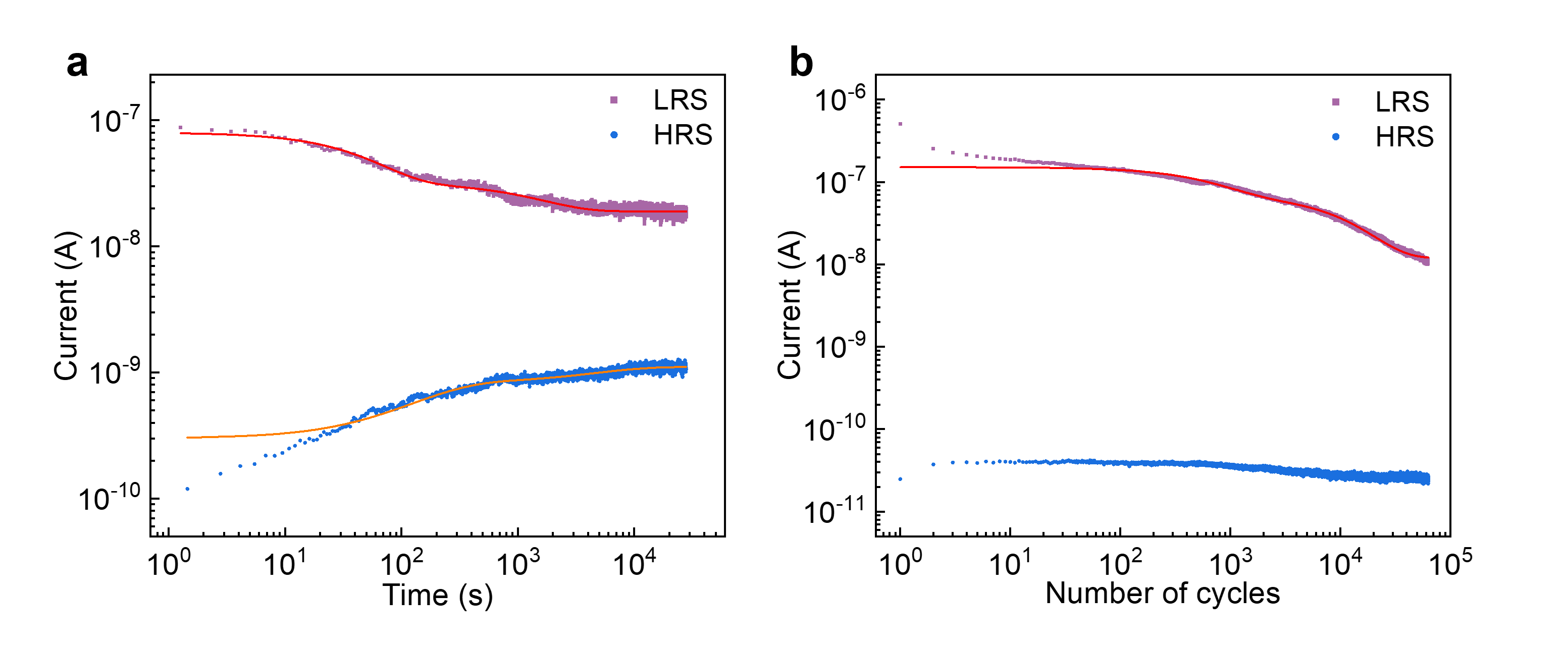


**Figure S8.** Fitted curves of a) retention and b) endurance characteristics.


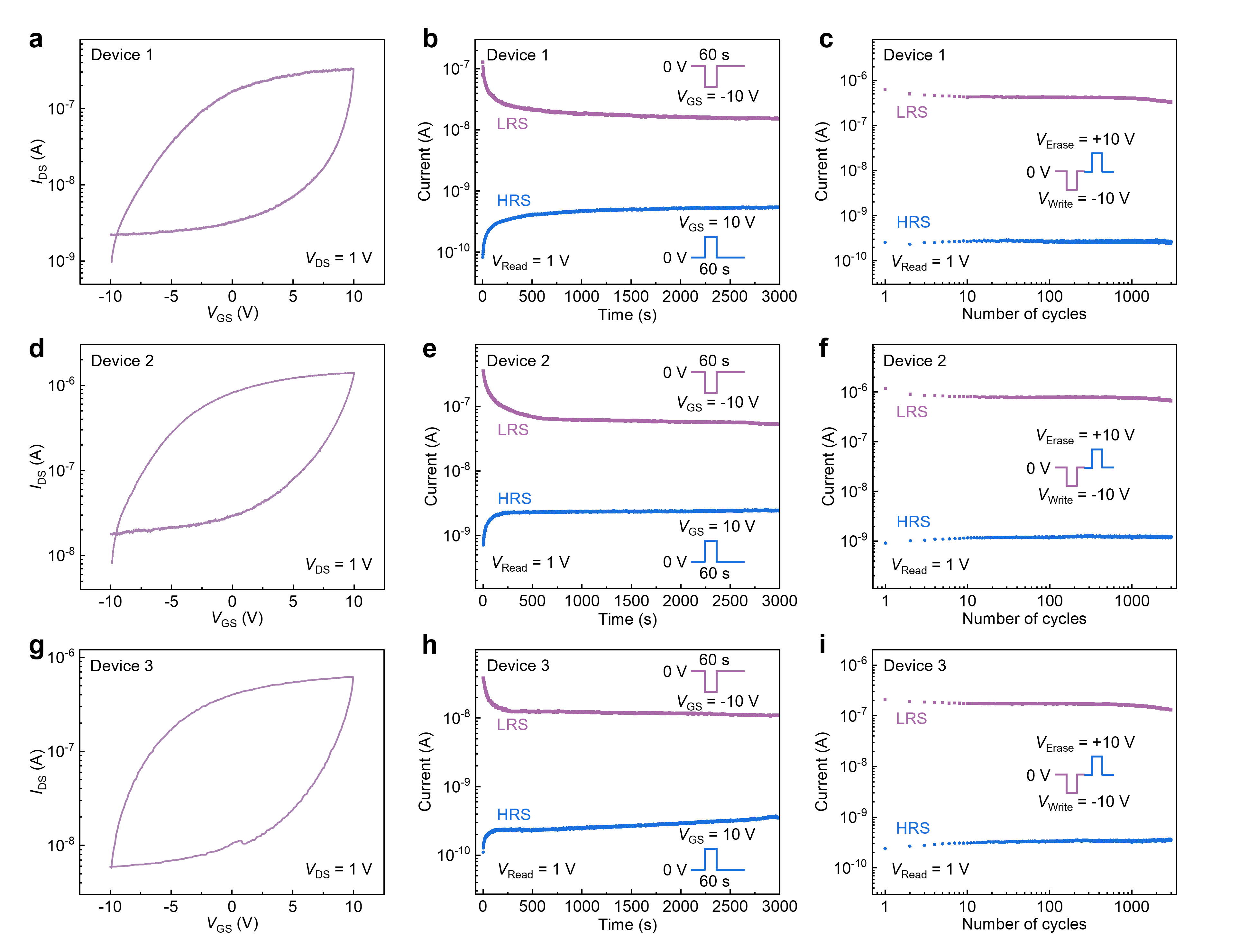


**Figure S9.** Device-to-device variations in a, d, g) transfer, b, e, h) retention, and c, f, i) endurance characteristics.


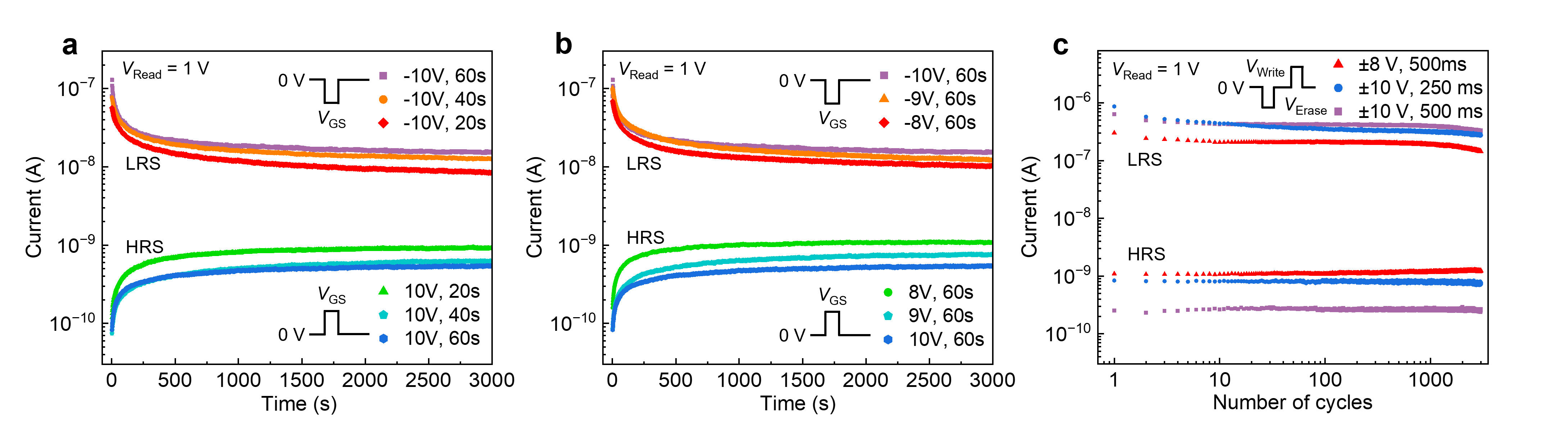


**Figure S10.** The retention characteristics of the same secure transistor under different a) pulse widths, b) pulse amplitudes. c) The endurance characteristics under different pulse amplitudes and widths.


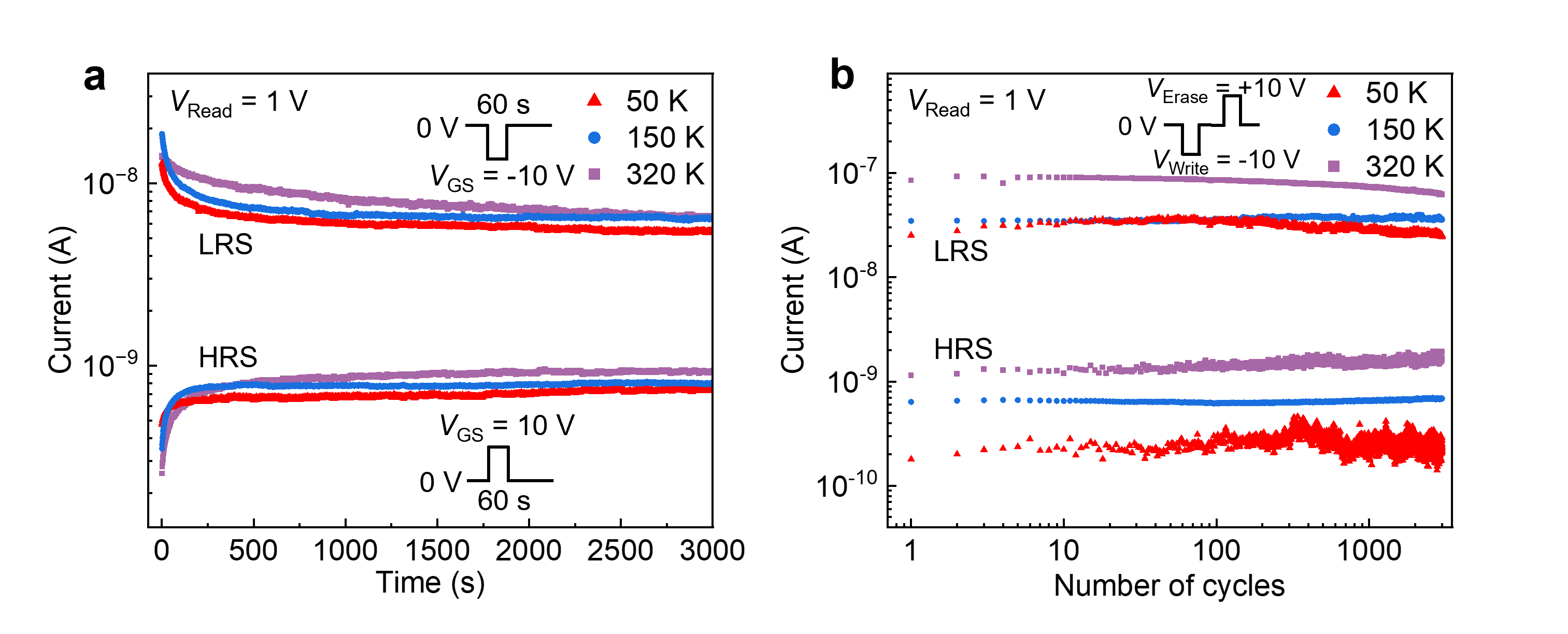


**Figure S11.** The a) retention and b) endurance characteristics of secure transistor at various temperatures.


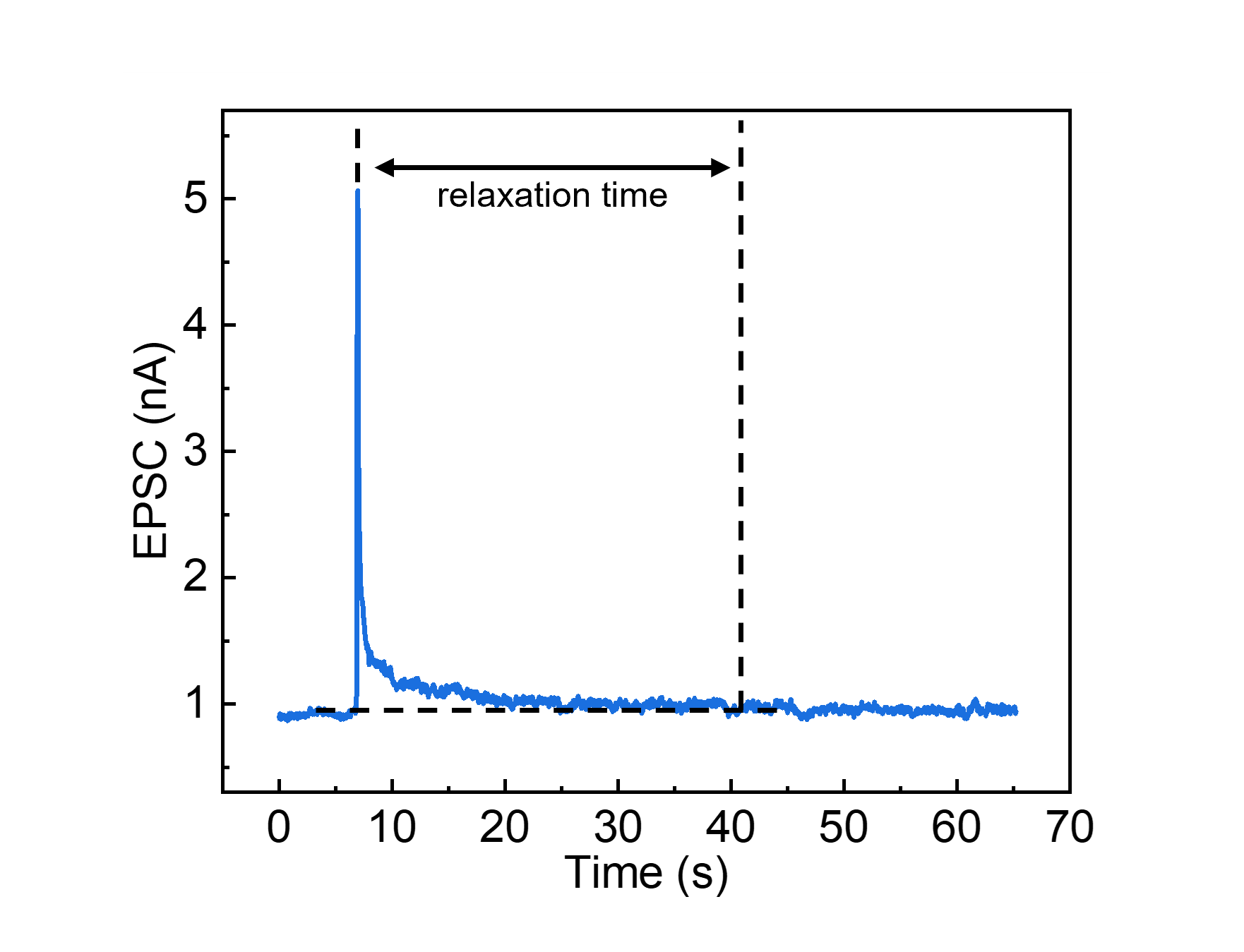


**Figure S12.** Statistical method of relaxation time of optoelectronic PSC.


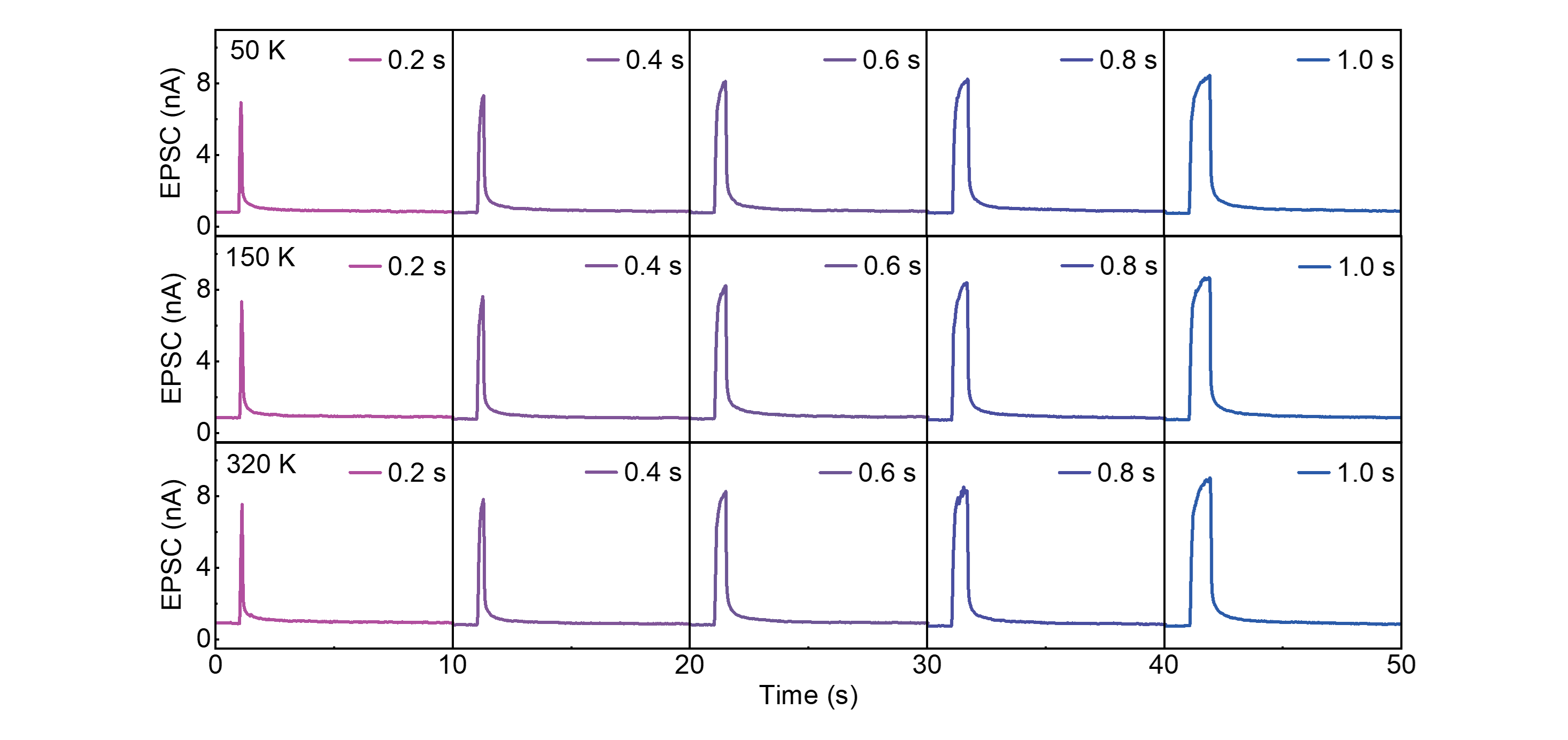


**Figure S13.** The photoresponse of the secure transistor at different temperatures with a fixed light intensity of 0.18 mW cm^-2^.


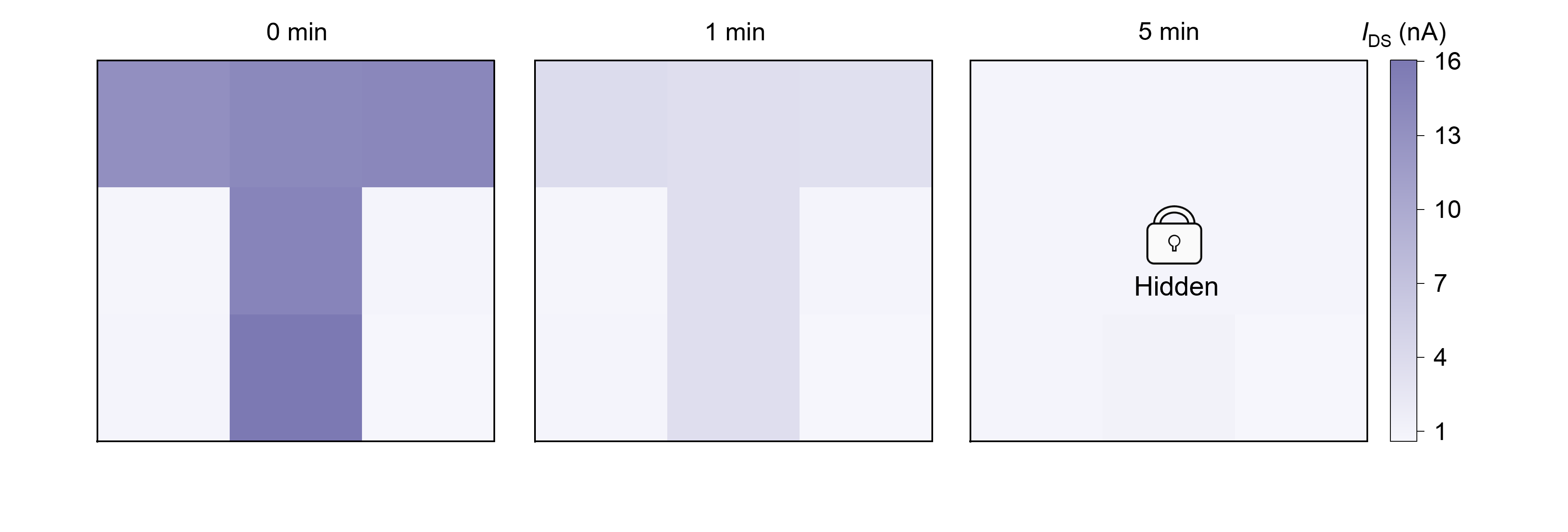


**Figure S14.** Images were captured at 0 min, 1 min, and 5 min after the removal of light stimulus and the watermark persists for more than 1 min. The light intensity in the letter region was set to 1.02 mW cm^-2^, with a pulse width of 1 s.


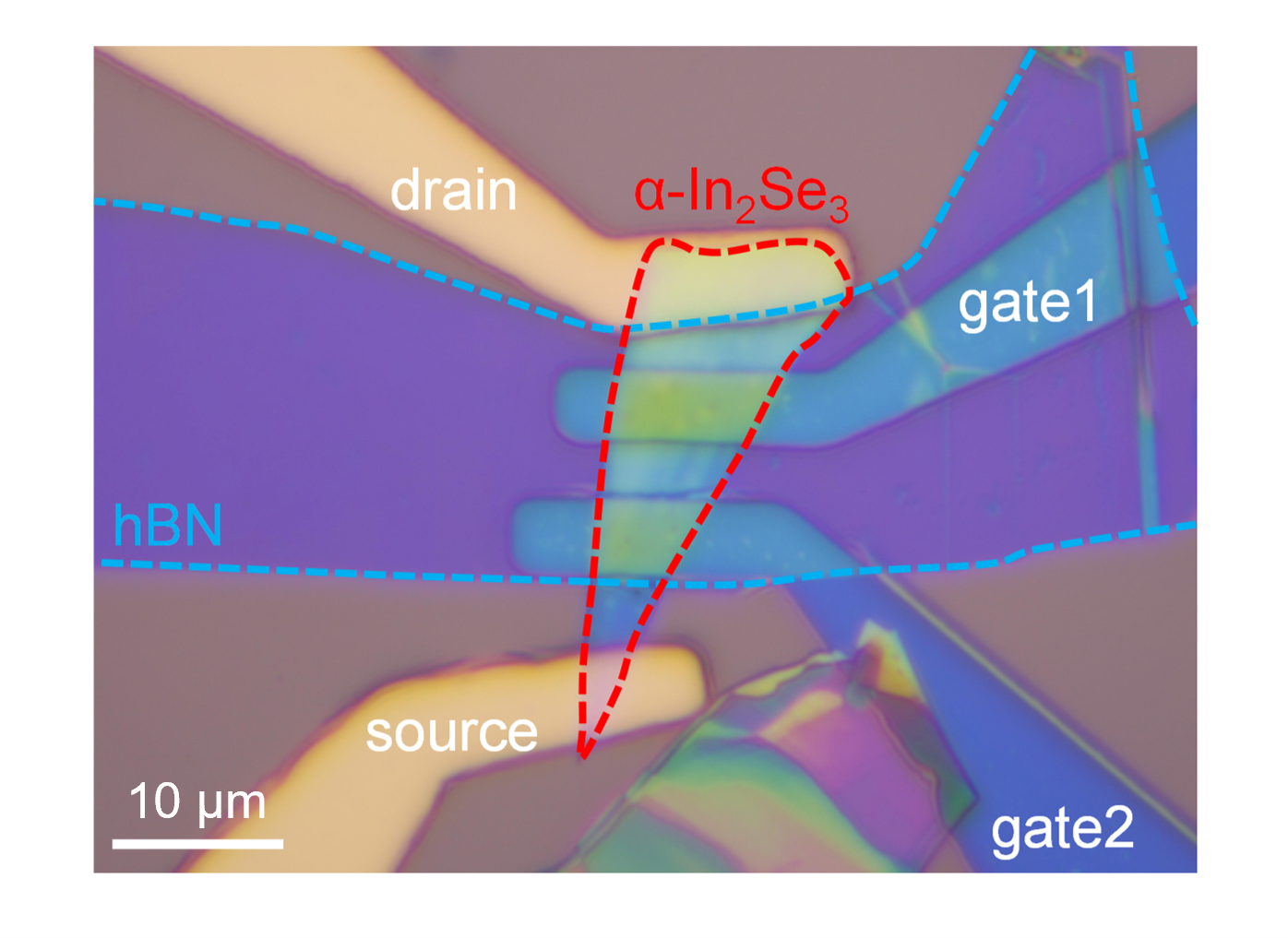


**Figure S15.** An optical microscope image of the split-gate device.
